# Supplementary material for: A prospective longitudinal study with treated hypertensive patients in Northern Bangladesh (PREDIcT-HTN) to understand uncontrolled hypertension and adverse clinical events: A protocol for 5-years follow-up
Source: PLoS One. 2022 May 31;17(5):e0269240. doi: 10.1371/journal.pone.0269240 (PMC9154182; doi:10.1371/journal.pone.0269240)
Supplement: Attachment — Submitted filename: Response to Reviewers.docx [file pone.0269240.s002.docx]

May 8, 2022

Professor Hafiz T.A. Khan, Ph.D., CStat

Academic Editor

PLOS ONE

**A prospective longitudinal study with treated hypertensive patients in Northern Bangladesh (PREDIcT-HTN) to understand uncontrolled hypertension and adverse clinical events: A protocol for 5-years follow-up**

Dear Professor Khan,

Thank you very much for your editorial suggestions and the reviewer’s comments. They were accommodating. I am attaching an itemized list of responses along with the revised manuscript.

In our response to the reviewer, we used bold font for the comments/questions by the referees and regular font for our answers, which are shown immediately following the questions/comments.

Thank you once again for the opportunity to submit a revised manuscript.

Ahmed Hossain, PhD

Professor, Department of Public Health

Director, Global Health Institute.

North South University.

**Reviewer #1:**

1. **Abstract:**

- **Method:**
  a. **What does it mean by “treated hypertensive patients”? A little more clarification in the abstract would be great.**
  b. **“The participant will be right censored if the patient develops MACE or death due to any cause, lost to follow-up or at the end of the study”. – Is this an extra “Or” in this sentence?**

**Authors:** Thank you very much for your comment. The term "treated hypertensive patients" refers to patients with hypertension receiving antihypertensive treatment. Patients are ignorant about their high blood pressure and thus do not take the necessary treatment in many instances. Other factors could include a poor socioeconomic situation, reliance on alternative medicine, and so on. However, the mentioned term is well-recognized in previous publications as well. A citation from a Q1 journal is given below:

R. Izzo *et al.*, “Development of Left Ventricular Hypertrophy in Treated Hypertensive Outpatients,” *Hypertension*, vol. 69, no. 1, pp. 136–142, Jan. 2017, doi: 10.1161/HYPERTENSIONAHA.116.08158.

On the second point, as MACE is the primary outcome, it has been separated with an extra "or". It appears that there is a grammatical mistake, which is corrected.

2. **Introduction:**

- **Why is this study important? We already know most of the risk factors of hypertension or CVD now. The authors can add some additional justifications in the Introduction - what is so unique about the study? And why do they want to conduct search research in Bangladesh?**

**Authors:** Thank you very much for your observations. The study's objective is to estimate the incidence of MACE and identify the associated factors related to uncontrolled hypertension. It is relevant because this information has never been investigated in northern Bangladesh, which partly represents the rural population of Bangladesh. The relationship between socio-demographic factors, anti-hypertensive compliance therapy, medication and dietary adherence, and hypertension control status would help identify implementation and policy gaps regarding lifestyle modification awareness and approaches. We are aware of the risk factors for hypertension and CVD; however, we do not intend to investigate them.

3. **Methods:**

- **Study design and setting:**
  a. **Is there any justification for – why you want to follow up with the enrolled participants 12 monthly? The gap between the follow-up periods is long, but there will also be more recall bias, death, and loss to follow-up (refuse, absent, etc.). How will you be going to manage all of these?**
  b. **Please mention in the write up clearly – who will be excluded from the study? Figure 1 has all the details, but better to mention them in the write-up.**

**Authors:** Thank you again for the comments. Even though the patients will be followed up annually as part of the trial, they will come to the center as their physician directs. In addition, the measures used to assess the quality of life, antihypertensive medication compliance, and nutritional adherence all involve recalling the previous seven days on the day of data collection. As a result, there is no issue with the follow-up interval. The duration of follow-up has less of an impact on loss to follow-up for comparative analysis. Additionally, the mechanism of re-inviting patients is described in detail. It also appears that the number of deaths will vary because of this gap.

The end of follow-up or the individuals who will be excluded is described in the "Yearly Follow-up Plan" section (Page 8, Line 208). Furthermore, the definition of MACE (major adverse cardiovascular event) is stated in the "Introduction" (Page 4, Line 74).

- **Rationale for planned sample size:
  a. What percentage did the authors consider for the loss to the follow-up group?**

**Authors:** Thank you very much for your comment. The required sample size is 768, whereas we aimed to recruit 2643. The planned sample size will have enough statistical power to meet any of the objectives of this study, even if the loss to follow-up rate is between 15-20% in each of the five visits. During the first follow-up, 2276 of the 2643 respondents visited the center, accounting for an about 14% non-response rate within the acceptable margin. This information is now added in the manuscript.

4. **Informed Consent form:**

- **Did the author plan to collect informed consent in every follow-up visit? If not, then the author should clearly mention the number of follow-up visits and how many years the participants will be followed-up?**

**Authors:** Thank you very much for your suggestion. We already have mentioned these in our informed consent form and took the consent.

------------------------------------------------------------------------------------------------------------------------------

**Reviewer #2:**

1. **There are a few instances where it is written that it will be done. However, what I believe is that these stages have already been done as per the time line set in the proposal. For example, in the abstract, it is written that the baseline data will be retrieved. Likewise, the participants have already given their consent and will not provide it. Please find these and correct them.**

**Authors:** Thank you very much for your observation. We have edited the manuscript accordingly.

2. **The first objective of the study stated must include the aim of investigating the 5-year incidence of MACE (Major Adverse Clinical Events).**

**Authors:** Thank you very much for this comment. We have included it in the manuscript.

3. **An additional value could be added by taking the rate of attrition (not having a final visit at 12 months) and rates of several categories of attrition (mortality, withdrawal from the study, transfer to non-study clinics, and loss to follow-up without identifiable cause) into consideration as a secondary outcome. Please include it in the analysis plan as well.**

**Authors:** Thank you very much for your suggestion. The attrition rate can be calculated as per our analysis plan; thus, we have included it in the paper.

4. **As per the follow-up design of the study, the death of the participant shall be recorded based on the death certificate or through telephonic conversation with the patient’s attendance. It might not be robust, but it will surely be a transparent and executable study plan.**

**Authors:** Thank you very much for such a detailed observation. We have corrected it accordingly.
